# Inverted T–shaped resection for midline anterior chest wall skin cancers: achieving oncologic clearance with primary closure

**DOI:** 10.1093/jscr/rjag364

**Published:** 2026-05-11

**Authors:** Nelson Buelvas

**Affiliations:** Department of Surgery, Universidad del Sinú, Cartagena, Colombia; Department of Breast Surgery and Soft Tissue Tumors, IMAT Oncomédica – Auna, Montería, Colombia

**Keywords:** skin cancer, squamous cell carcinoma, melanoma, surgical excision, chest wall reconstruction

## Abstract

Midline anterior chest wall skin cancers may pose a reconstructive challenge due to limited presternal skin laxity. Conventional elliptical excision using a 3:1 length-to-width ratio can result in excessive cranio-caudal scar extension toward the cervical or upper abdominal regions. We report two cases of midline anterior chest wall skin cancers—a nodular melanoma and an extensive squamous cell carcinoma—managed using an inverted T–shaped resection design. Oncologic margins were planned according to histologically confirmed diagnoses, followed by en bloc excision and bilateral local tissue advancement to achieve primary closure. In both cases, complete tumor excision with negative margins was achieved without postoperative complications. This inverted T–shaped resection represents a simple and reproducible surgical approach that facilitates primary closure while preserving oncologic principles in selected midline anterior chest wall skin cancers.

## Introduction

Skin malignancies of the anterior chest wall are frequently encountered in surgical practice [1], particularly in areas of chronic sun exposure such as the presternal region. While small lesions located in the upper chest can often be managed with standard elliptical excision and primary closure [2], larger tumors or those located along the midline pose a distinct reconstructive challenge due to limited skin laxity and the aesthetic and functional consequences of elongated closures [3].

**Figure 1:**
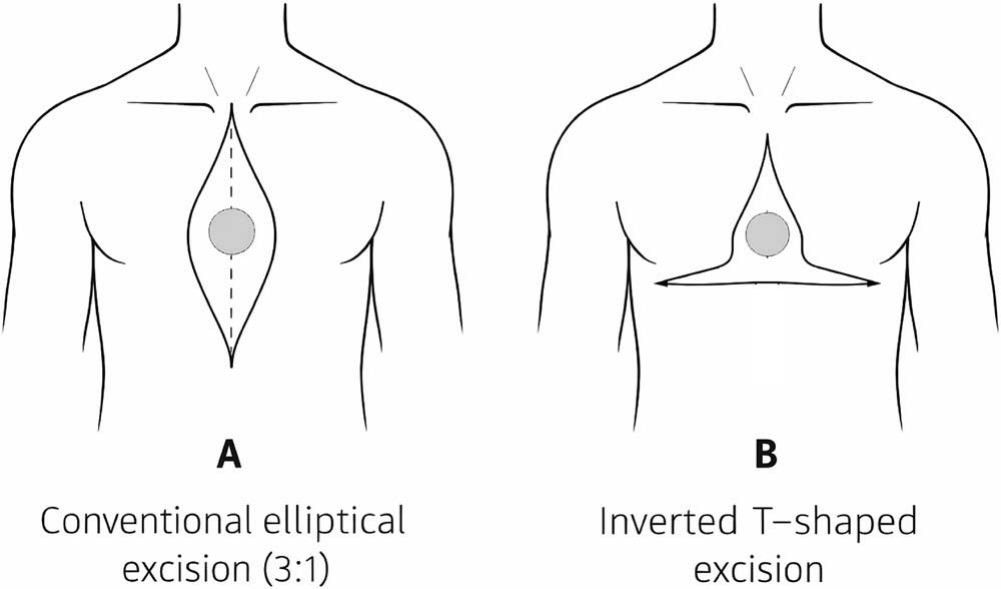
Schematic comparison of excision designs for midline anterior chest wall skin tumors. (A) Conventional elliptical excision using a 3:1 length-to-width ratio, which may result in excessive cranio-caudal extension of the scar toward the cervical region or upper abdomen. (B) Inverted T–shaped excision design, allowing redistribution of closure vectors and containment of the surgical scar within the thoracic region.

The midline anterior chest wall, especially overlying the sternum, is characterized by relatively inelastic skin and restricted tissue mobility [4]. In this setting, conventional elliptical excision using a 3:1 length-to-width ratio may result in excessively long vertical scars extending toward the cervical or upper abdominal regions, potentially leading to patient dissatisfaction, wound tension, or delayed healing (Fig. 1). Alternative reconstructive strategies, including skin grafting or regional flap reconstruction, may achieve coverage but often at the cost of inferior aesthetic outcomes, increased operative complexity or the need for additional reconstructive expertise [5].

Despite the frequency of midline anterior chest wall skin tumours, there is limited literature addressing modifications of the excision design itself as a means to facilitate oncologically sound resection while allowing primary closure with adjacent local tissue. Most descriptions of inverted T–shaped incisions are confined to breast surgery, where the design serves a fundamentally different reconstructive purpose [6, 7]. Its application as a resection pattern for cutaneous malignancies of the anterior chest wall has not been widely described.

The aim of this report is to describe an inverted T–shaped resection design for selected midline anterior chest wall skin cancers, allowing adequate oncologic excision while facilitating tension-free primary closure through bilateral local tissue advancement.

## Case report

### Surgical technique

After histologic confirmation of malignancy by punch biopsy, resection margins were planned according to tumor type and established oncologic guidelines. Patients were positioned supine, and the lesion was centered within the planned excision.

An inverted T–shaped resection pattern was designed, with the vertical component centered over the tumor and the horizontal limb oriented along the inframammary fold as an anatomic reference. Following en bloc excision of the skin and subcutaneous tissue down to the prepectoral fascia, bilateral fasciocutaneous advancement flaps were elevated in the prepectoral plane to ensure adequate thickness and reliable perfusion through the lateral thoracic system and intercostal perforators. As expected, perforators from the internal mammary system were necessarily divided during the central oncologic resection.

An initial anchoring suture was placed at the junction of the horizontal and vertical components of the incision, fixing the medial edges of the advancement flaps to the central portion of the horizontal limb. This maneuver facilitated global wound alignment and redistribution of closure tension. The remaining closure was completed in layers, and metallic skin staples were used to provide prolonged tensile strength, particularly given the advanced age and skin fragility of the patients.

### Case 1

A 71-year-old male patient from a rural area of the Colombian Caribbean region presented with a pigmented lesion located along the midline of the anterior chest wall. A punch biopsy confirmed the diagnosis of nodular melanoma. Preoperative clinical examination and ultrasound suggested a possible satellite lesion cranial to the primary tumor.

Wide local excision was planned according to melanoma-specific oncologic guidelines. Intraoperatively, the suspected satellite lesion corresponded to a benign prominence caused by malunited costal fractures, with no tumour involvement.

**Figure 2:**
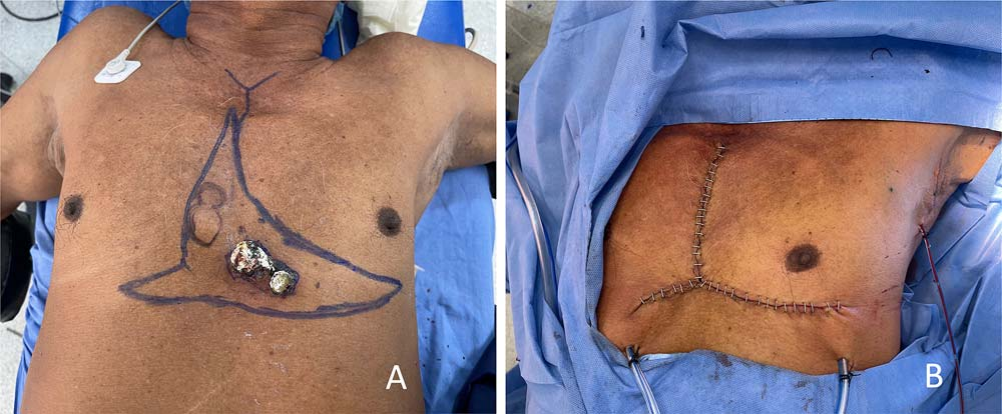
Case 1 (nodular melanoma). (A) Preoperative clinical appearance of a midline anterior chest wall melanoma, with the planned inverted T–shaped resection design outlined to achieve adequate oncologic margins. (B) Immediate postoperative appearance following en bloc resection and closure using bilateral pectoral fasciocutaneous advancement, resulting in a contained inverted T–shaped scar within the thoracic region.

Histopathological examination revealed a nodular melanoma measuring 2.5 × 2 cm, with a Breslow thickness of 7 mm and ulceration. Microsatellitosis was present, while mitotic activity, lymphovascular invasion, and tumour regression were absent. All surgical margins were free of tumor. The postoperative course was uneventful, and the patient was discharged on postoperative day 1 (Fig. 2).

### Case 2

An 87-year-old male patient from a rural area of northern Colombia presented with an extensive cutaneous lesion involving the midline anterior chest wall. His medical history included multiple prior cutaneous squamous cell carcinomas and a previous radical neck dissection. A punch biopsy confirmed cutaneous squamous cell carcinoma.

**Figure 3:**
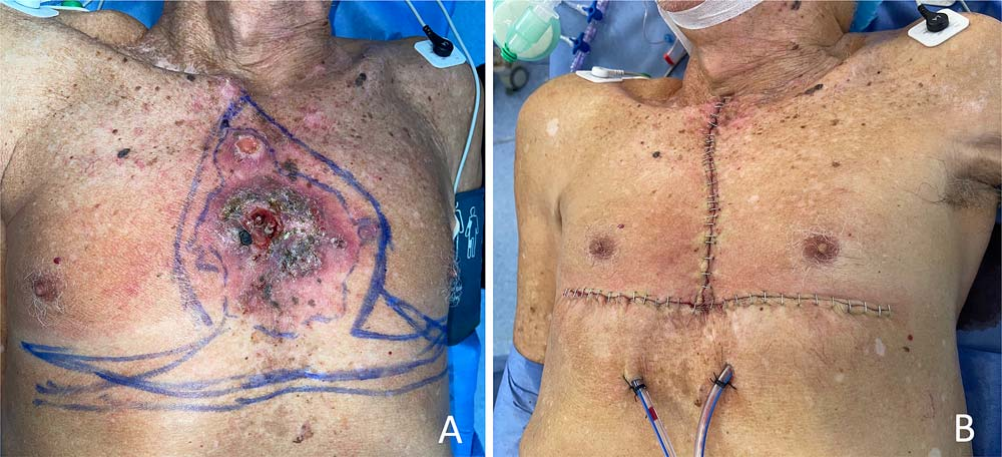
Case 2 (extensive squamous cell carcinoma). (A) Preoperative clinical appearance of a large, ulcerated, and exophytic squamous cell carcinoma involving the midline anterior chest wall, in the setting of diffuse actinic damage. (B) Immediate postoperative appearance after wide local excision and closure using the inverted T–shaped resection design, achieving complete defect coverage and preservation of anterior chest wall contour despite the extensive tumor size.

Histopathological examination of the surgical specimen revealed an infiltrating, keratinizing squamous cell carcinoma measuring 13.1 × 9.7 cm, moderately differentiated, with a maximum depth of invasion of 2 cm extending into the hypodermis. Lymphovascular invasion was present, while perineural invasion was absent. All peripheral and deep margins were free of invasive carcinoma; the superior margin showed actinic keratosis without invasive tumour involvement. The postoperative course was uncomplicated, and the patient was discharged on postoperative day 1 (Fig. 3).

## Results

Complete en bloc tumor excision with adequate oncologic margins was achieved in both cases. Primary closure using bilateral local tissue advancement was successful in all patients.

In the melanoma case, final pathology confirmed high-risk features with all margins free of tumor. In the squamous cell carcinoma case, histopathology demonstrated a large, deeply infiltrating tumor with lymphovascular invasion, yet all margins remained free of invasive disease. No postoperative complications occurred, and early wound healing was satisfactory in both patients.

## Discussion

Surgical management of midline anterior chest wall skin cancers is challenging due to limited presternal skin laxity [8]. Conventional elliptical excision may result in excessive cranio-caudal scar extension and increased wound tension, particularly for larger or deeply infiltrating tumors [4].

The inverted T–shaped resection described here emphasizes modification of the excision design itself rather than reliance on complex reconstruction. By redistributing closure vectors, this approach allows tension-free primary closure while maintaining oncologic integrity [9, 10].

In many low- and middle-income settings, patients frequently present with advanced or ulcerated cutaneous malignancies [11, 12]. Beyond barriers to healthcare access, delayed presentation may be influenced by limited health literacy and sociocultural factors, as early lesions are often mistaken for benign conditions [13]. In this context, pragmatic surgical strategies that permit adequate oncologic resection while minimizing reconstructive complexity are particularly valuable.

Limitations of this report include the small number of cases and short follow-up. Nevertheless, this technique represents a reproducible and anatomically rational option for selected midline anterior chest wall skin cancers.
